# Supplementary material for: Microgeographic variation in locomotor traits among lizards in a human-built environment
Source: PeerJ. 2016 Mar 10;4:e1776. doi: 10.7717/peerj.1776 (PMC4793326; doi:10.7717/peerj.1776)
Supplement: Supplementary file 1 [file peerj-04-1776-s005.docx]

Supplemental Table 1: Results of correlations between three morphometrics and four performance traits. All morphometrics and performance measures were Log10 transformed to meet asusmptions of normality.

		**Velocity over Rock**		**Velocity over Sand**		**Acceleration over Rock**		**Acceleration over Sand**	
		Males	Females	Males	Females	Males	Females	Males	Females
**SVL**	*p*=	0.7932	0.0815	0.2963	0.984	0.7744	0.8981	0.8505	0.8783
	R^2Adj=	-0.0055	0.0144	0.0005	-0.007	-0.0055	-0.0069	-0.0059	-0.007
**Length of longest toe**	*p*=	0.3131	0.8521	0.9993	0.2372	0.5383	0.9209	0.5549	0.6911
	R^2Adj=	0.0001	-0.0068	-0.006	0.0029	-0.0037	-0.007	-0.004	-0.006
**Total hindlimb length**	*p*=	0.3066	0.837	0.4916	0.6394	0.3601	0.7348	0.8442	0.9921
	R^2Adj=	0.0003	-0.00679	-0.0032	-0.0056	-0.0009	-0.006	-0.0059	-0.0072
